# Occurrence, Serotypes and Virulence Characteristics of Shiga-Toxin-Producing *Escherichia coli* Isolates from Goats on Communal Rangeland in South Africa

**DOI:** 10.3390/toxins14050353

**Published:** 2022-05-18

**Authors:** Mogaugedi N. Malahlela, Beniamino T. Cenci-Goga, Munyaradzi C. Marufu, Thierry Y. Fonkui, Luca Grispoldi, Eric Etter, Alan Kalake, Musafiri Karama

**Affiliations:** 1 Veterinary Public Health Section, Department of Paraclinical Sciences, Faculty of Veterinary Science, University of Pretoria, Onderstepoort 0110, South Africa; u28556021@tuks.co.za (M.N.M.); beniamino.cencigoga@unipg.it (B.T.C.-G.); youmbifonkui@yahoo.com (T.Y.F.); 2 Departiment of Veterinary Medicine, Laboratorio di Ispezione Degli Alimenti di Origine Animale, University of Perugia, 06126 Perugia, Italy; grisluca@outlook.it; 3 Department of Veterinary Tropical Diseases, Faculty of Veterinary Science, University of Pretoria, Onderstepoort 0110, South Africa; chris.marufu@up.ac.za; 4 Department of Production Animal Studies, Faculty of Veterinary Science, University of Pretoria, Onderstepoort 0110, South Africa; eric.etter@up.ac.za; 5 Centre de Coopération Internationale en Recherche Agronomique Pour le Développement (CIRAD), Unité Mixte de Recherche (UMR), Animal Santé, Territoires, Risques et Ecosystèmes (ASTRE), 97170 Petit Bourg, France; 6 ASTRE, University Montpellier, CIRAD, INRAE, France Baillarguet International Campus, University of Montpellier, 34070 Montpellier, France; 7 Gauteng Department of Agriculture and Rural Development, Johannesburg 2001, South Africa; alan.kalake@gauteng.gov.za

**Keywords:** goats, STEC, serotypes, virulence, South Africa

## Abstract

Shiga-toxin-producing *Escherichia coli* is a foodborne pathogen commonly associated with human disease characterized by mild or bloody diarrhea hemorrhagic colitis and hemolytic uremic syndrome. This study investigated the occurrence of STEC in fecal samples of 289 goats in South Africa using microbiological culture and PCR. Furthermore, 628 goat STEC isolates were characterized by serotype (O:H) and major virulence factors by PCR. STEC was found in 80.2% (232/289) of goat fecal samples. Serotyping of 628 STEC isolates revealed 63 distinct serotypes including four of the major top seven STEC serogroups which were detected in 12.1% (35/289) of goats: O157:H7, 2.7% (8/289); O157:H8, 0.3%, (1/289); O157:H29, 0.3% (1/289); O103:H8, 7.6% (22/289); O103:H56, 0.3% (1/289); O26:H2, 0.3% (1/289); O111:H8, 0.3% (1/289) and 59 non-O157 STEC serotypes. Twenty-four of the sixty-three serotypes were previously associated with human disease. Virulence genes were distributed as follows: *stx*1, 60.6% (381/628); *stx*2, 72.7% (457/628); *eaeA*, 22.1% (139/628) and *hlyA*, 78.0% (490/628). Both *stx*1 and *stx*2 were found in 33.4% (210/628) of isolates. In conclusion, goats in South Africa are a reservoir and potential source of diverse STEC serotypes that are potentially virulent for humans. Further molecular characterization will be needed to fully assess the virulence potential of goat STEC isolates and their capacity to cause disease in humans.

## 1. Introduction

Shiga-toxin-producing *E. coli* (STEC) is a foodborne pathogen commonly associated with enteric disease in humans characterized by mild watery or bloody diarrhea hemorrhagic colitis (HC) and the hemolytic uremic syndrome (HUS) as a complication in 5–10% of humans. According to the Foodborne Disease Burden Epidemiology Reference Group (FERG), STEC was responsible for around 2.5 million of new cases of human disease, of which 1.2 million may have been foodborne, with 3330 HUS cases and 269 deaths, which corresponded to 27,000 disability-adjusted life years (DALYs) in 2010 [1].

Domestic ruminants including cattle, sheep, and goats are the main reservoirs of STEC [2,3,4,5]. Ingestion of contaminated meat, dairy products, vegetables, and water is a risk factor for acquiring STEC infection in humans [2,6,7]. Furthermore, contact with animals carrying STEC has also been associated with disease in humans [8,9].

More than 1000 different serotypes of Shiga-toxin-producing *Escherichia coli* (STEC) have been described in humans, animals and the environment [10,11,12]. STEC O157:H7 was the first serotype to be associated with a human disease outbreak and remains the most frequent strain in human illness [13]. However, numerous non-O157 STEC serotypes have also been linked to outbreaks and severe disease in humans including HUS [10,11,12,14,15]. STEC O26, O45, O103, O111, O121 and O145 are the six most frequently incriminated non-O157 serogroups in human disease [14,15]. Together with STEC O157, these “top 6” non-O157 serogroups are the major seven STEC serogroups, also colloquially termed “Big 7 or Top 7” STEC. Furthermore, according to the Centers for Disease Control and Prevention (CDC), at least 13 serotypes associated with top 6 STEC serogroups including O26:H11 or nonmotile (NM); O45:H2 or NM; O103:H2, H11, H25; or NM; O111:H8 or NM; O121:H19 or H7; and O145: NM may be responsible for up to 80% of cases of human STEC disease in the United States [14]. In South Africa, serotypes O26:H11, O111:H8, O157:H7 and O107/O117:H7 were frequently implicated in disease between 2006–2013 [16].

Bacteriophage-encoded Shiga toxins (*stx*1 and *stx*2) and a number of *stx* subtypes are considered the major STEC virulence factors [17,18,19,20]. Identification of STEC is based on detection of one or more Shiga-toxin-encoding genes (*stx*1 and *stx*2) [17,19,20]. STEC that carry *stx*2 are more frequently associated with severe disease including HUS in comparison to strains that possess *stx*2 alone or both *stx*1 and *stx*2 concomitantly [21,22,23,24,25]. 

Intimin (*eaeA*) is an additional important STEC virulence factor [26,27]. The gene encoding intimin is located on a 35 Kb pathogenicity island in *E. coli* O157:H7 termed the locus of enterocyte effacement (LEE) [28,29]. Intimin is responsible for intimate adherence of STEC to intestinal epithelial cells and formation of typical attaching and effacing (A/E) lesions in the intestine characterized by actin-rich pedestals and loss of brush border microvilli under bound bacteria [30]. Furthermore, STEC possess plasmid-encoded virulence markers including a hemolysin (*hly*A) and additional virulence-associated genes which are located on pathogenicity-islands [31,32,33,34,35]. 

Current reports on the occurrence and characteristics of STEC in animals and humans in South Africa are scanty. Furthermore, the few studies that have reported on the occurrence of STEC in South Africa have largely investigated the presence of STEC in cattle populations [36,37,38] while studies on the prevalence of STEC in other ruminants including goats are lacking. Therefore, the main objectives of this study were (1) to determine the occurrence of STEC in goats raised on communal rangeland in South Africa and (2) characterize STEC by serotype (O:H) and major virulence factors (*stx*1*, stx*2, *eaeA*, and *hlyA*). The overall goal is to contribute to STEC monitoring and surveillance in South Africa. 

## 2. Results

### 2.1. STEC Occurrence 

**Figure 1:**
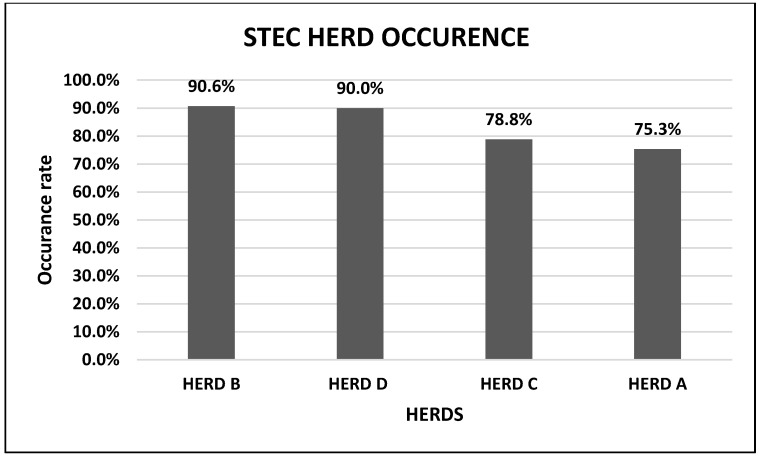
STEC Occurrence within goat herds.

A total of 289 fecal samples were collected from four goat herds on communal rangeland (herd A, B, C, and D) in the Gauteng province of South Africa and screened for STEC. PCR revealed that 80.2% (232/289) of goat fecal samples were positive for STEC. STEC was detected in 75.3% (116/154) of goats in herd A; 90.6% (39/43) in herd B; 78.8% (41/52) in herd C and 90% (36/40) in herd D (Table 1 and Figure 1). 

### 2.2. STEC O:H Serotypes

**Figure 2:**
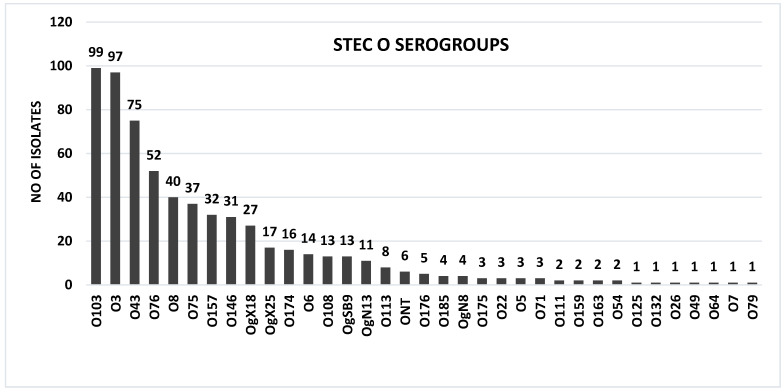
Distribution of STEC O serogroups.

**Figure 3:**
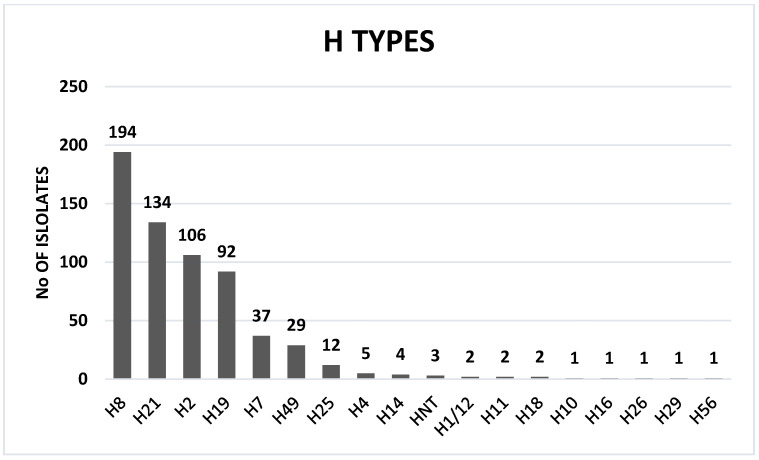
Distribution of STEC H-types.

At least 99% (622/628) of isolates were O:H serotypeable by PCR. PCR serogrouping revealed 34 O groups and 17 H types with a total of 63 O:H distinct serotypes. Six isolates were O-untypable (ONT) (Figure 2 and Table 1) and three H-untypable (HNT) (Figure 3 and Table 1). The highest number of STEC serotypes was recovered from herd A—41 serotypes; followed by herd B—21 serotypes; herd C—10 serotypes; and herd D—8 serotypes. STEC O76:H19 was recovered from all herds: STEC O146:H21 and OgX18:H2 were recovered from herd A, C, and D. STEC O8:H14, OgSB9:H19 and OgX25:H8 were found in herd A and B. STEC O43:H2, O43:H8, O113:H8 and O157:H7 were recovered from herd A and C. STEC O3:H21 and O8:H19 were recovered from herd A and D. 

Among the 34 O serogroups, 18 were associated with a single H type (O5:H19, O7:H7, O22:H8, O26:H2, O49:H11, O64:H18, O75:H8, O79:H8, O111:H8, O113:H8, O125:H19, O132:H8, O146:H21, O174:H8, O176:H4, O185:H8, ON8:H7, OX25:H8), and 16 O groups (O3, O6, O8, O43, O54, O71, O76, O103, O108, O157, O159, O163, O175, ON13, OSB9 and OX18) were associated with more than one H type (Supplementary Table S1). The following 17 H types were detected: H1/12, H2, H4, H7, H8, H10, H11, H14, H16, H18, H19, H21, H25, H26, H29, H49 and H56 (Figure 3). The distribution of different STEC serotypes per goat herd is shown in Table 1. 

Among the 63 O:H distinct serotypes, 55.5%, (35/63) were each represented by a single isolate while the remaining 44.4% (28/63) were represented by more than one isolate (Table 1). O:H serotype combinations can be found in Supplementary Table S1. 

The six most frequent goat STEC serotypes were: O3:H21, 7.9% (23/289); O103:H8, 7.6% (22/289); O43:H2, 6.5% (19/289); O76:H19, 5.8% (17/289); O75:H8, 3.1%, (9/289); O157:H7, 2.7% (8/289). Big seven STEC serotypes were recovered from 11.0% (32/289) of goats. Big seven STEC serotypes were distributed as follows among goats: O157:H7, 2.7% (8/289); O103:H8, 7.6% (22/289); O26:H2, 0.3% (1/289); O111:H8, 0.3% (1/289); O103:H56, 0.3% (1/289); O157:H8, 0.3% (1/289); O157:H29, 0.3% (1/289).

### 2.3. STEC Virulence Characteristics 

The distribution of four STEC virulence genes among the 628 STEC isolates was as follows: *stx*1*,* 60.6% (381/628); *stx*2, 72.7% (457/628); *eaeA*, 22.1% (139/628); *hlyA*, 78.0% (490/628). Both *stx*1 and *stx*2 were found concomitantly in 33.4% (210/628) of isolates (Supplementary Table S2). The following major gene combinations were observed: *stx*1 *stx*2 *hlyA*, 26.9%, (169/628); *stx*2 *eaeA hlyA* 20.3%, (128/628) and *stx*1 *hlyA* 20.0%, (126/628). STEC characteristics are depicted in Supplementary Table S2. 

The *eaeA* gene was observed in 22.1%, (139/628) of isolates which corresponded to 12.8% (37/289) of goats which were *eaeA* positive. Of the 139 isolates that were *eaeA* positive, 131 belonged to five of the major seven serogroups including O157:H7 (30/628 isolates, 8/289 goats), O157:H8 (1/628 isolates, 1/289 goats), O103:H8 (97/628 isolates, 22/289 goats), O26:H2 (1/628 isolate, 1/289 goats) and O111:H8 (2/628 isolates, 1/289 goats). In addition, eight isolates (1.2%) which were non-Big seven STEC serotypes possessed *eaeA*: O71:H14 (1/628 isolate, 1/289 goats), O108:H25 (6/628 isolates, 2/289 goats), O163:H8 (1/628 isolate, 1/289 goats) were also *eaeA*-positive (1.2%, 8/628). Most of the *eaeA* positive isolates, 92.0% (128/139) had the *stx*2*eaeA* genotype (O71:H14, O103:H8, 157:H7, O157:H8) while the remaining 7.9% (11/139) isolates (O26:H2, O163:H8, O103:H8, O108:H25 and O111:H8) were *stx*1*eaeA* positive. 

## 3. Discussion

Previous reports from different countries have shown that goats are a reservoir of STEC [4,39,40,41,42]. Furthermore, contact with goats and food products of goat origin have been associated with STEC disease in humans [4,43]. However, published reports on the occurrence and characteristics of STEC in goats are few in comparison to cattle and sheep. Furthermore, reports on the occurrence of STEC in goats in South Africa are non-existent. This study investigated the occurrence of STEC and characterized STEC isolates in four separate goat herds in South Africa. The overall occurrence of STEC in the goat populations surveyed was 80.2% (232/289). The occurrence of STEC in this study was very high in comparison to similar studies in Germany [42,44], Brazil (57.5%) [40], Spain (47.7%) [39,45], Vietnam (31.5%) [41] and Bangladesh (11.8%) [46] which reported STEC detection rates ranging from 11.8% to 75.3% in goats. Other reports have found STEC occurrence rates ranging from 23.9% to 89.3% in different countries, but these studies were conducted on far smaller goat sample populations (≤46) to warrant a valid comparison with the present study [47,48,49,50]. 

The within-herd occurrence of STEC ranged from 75.3% (116/154) to 90.6% (39/43) which was significantly higher in comparison to similar studies in Brazil (46.7–73.3%) [40] and Vietnam (15–65%) [41]. Moreover, all the four goat herds were positive for STEC, in agreement with similar reports elsewhere [40,41,42]. However, the number of goat samples which were tested per herd in this study was “significantly” higher compared to the reports from Brazil (106), Vietnam (205) and Germany (93) which may explain why the within-herd STEC occurrence in this study was also higher. The higher occurrence of STEC in goats in this study may be ascribed to higher shedding of STEC in the goat population studied, variations in geographic locations, age (kids vs. adults), goat diet (grazing or browsing vs. concentrate), and management practices. Furthermore, the use of a suitable enrichment broth and two selective and sensitive STEC culture and isolation media may have increased STEC recovery [51,52,53,54,55,56]. 

In the present study, 99.0% of goat STEC isolates were serotypeable by PCR. A total of 63 serotypes (34 O and 17 H groups) were recovered from goats. The number of serotypes detected in this study was very high compared to previous studies [39,40,45]. The recovery of a very high number of serotypes may also be ascribed to the high shedding of STEC in the goat populations tested. Furthermore, the use of a sensitive, specific, accurate and reliable PCR protocol for O:H serotyping may have led to the identification of more serotypes than usually found with traditional serotyping [57,58,59]. Furthermore, PCR O:H serotyping has the advantage of detecting O-untypable (OUNT) and H-nontypeable and/or non-motile (HNT/NM) *E. coli* isolates that carry genes encoding O:H antigens but cannot be expressed. In this study, we were able to validate the Iguchi et al. [58,59] and Banjo et al. [57] *E. coli* PCR serotyping (O:H) protocols which were highly discriminatory and unambiguously serotyped the large number of goats STEC isolates tested in this study [57,58,59]. To our knowledge, this is the most extensive serotyping of goat STEC isolates, worldwide. 

Among the 63 serotypes, only 4 serotypes belonged to the major 7 STEC serogroups. STEC O103:H8 (15.6%) was the most frequent Big seven STEC among goats, followed by STEC O157:H7 (4.7%), O111:H8 and STEC O26:H2. Overall, the major seven STEC serotypes accounted for 21.3% of all isolates which were serotyped, in contrast to most similar studies which never recovered major seven STEC from goats [39,40,43,44,45,60,61]. However, Schilling et al. [48] found a higher proportion of top seven STEC, although the recovered serotypes were those which have never been reported in human disease, in contrast to our results which showed that most of the top seven serotypes we recovered were previously incriminated in human disease outbreaks except for STEC O157:H29, O103:H8 and O103:H56. 

Previously, STEC O157:H7 has been incriminated in foodborne disease after consumption of raw goat milk and home-made cheese made from raw milk [43,62,63]. Furthermore, STEC O157 and STEC O103 have been incriminated in human disease after contact with goats in the USA [8] while sources other than goats have frequently associated STEC O157:H7, O111:H8 and O26:H2 to human disease worldwide including South Africa [16]. According to the STEC seropathotype classification, STEC O157:H7 is considered a seropathotype A strain, frequently incriminated in outbreaks and severe human disease while O111:H8 and O26:H2 are moderately implicated in outbreaks and less frequent in severe human disease, in comparison to STEC O157:H7 [32]. However, in this study most major seven STEC isolates were classified as STEC O103:H8. Previously, STEC O103:H8 was isolated from healthy goats and calves in China and Argentina, respectively [64,65]. In addition, only one study has reported the recovery of STEC O103:H8 from patients and asymptomatic food handlers in Japan [66]. However, this study never specified whether the STEC O103:H8 isolate was from patients or asymptomatic food handlers [66]. Therefore, although O103:H8 is classified as a major STEC (serogroup), its importance as a human pathogen remains unclear as there are no reports until now which have unequivocally associated this STEC serotype with human disease. 

The remaining 59 serotypes were non-O157, of which 24 have been previously incriminated in mild to severe human disease worldwide including South Africa, Europe, North America and Asia [10,11,12,16,67]. The recovery of STEC serotypes which have been associated with mild to severe human disease is evidence that goats are a reservoir and a potential source of these highly pathogenic STEC strains in South Africa. 

Highly diverse and farm specific STEC serotypes were observed in individual goat herds except for STEC O76:H19 which was the serotype shared among the four goat herds surveyed while STEC O146:H21 and OgX18:H2 were recorded in three herds. Overall, the highly diverse and farm specific serotypes are most likely a reflection of the fact that the four herds were situated in geographically separate and distant areas from each other to allow isolate interchange between herds. 

Regarding the virulence characteristics of the STEC isolates under study, *stx*2 was more frequent that *stx*1 among goat STEC in contrast to similar studies which have shown that *stx*1 is predominant among goat STEC isolates [39,41,42,45,50,68,69,70,71]. However, our findings agree with a study by Oliveira et al. [40] which reported that *stx*2 was more prevalent in goat STEC isolates. Reports on clinical STEC have suggested that *stx*2-positive isolates are more virulent and frequently incriminated in severe human disease including hemorrhagic colitis and hemolytic uremic syndrome in comparison to STEC isolates carrying *stx*1 or both *stx*1 and *stx*2 [21,22,23,24,25].

The *hlyA* gene was present in 78.0% (490/628) of goat STEC isolates, consistent with previous reports which have shown similar rates in goat STEC elsewhere [39,42,44]. However, lower rates of *hlyA* ranging from 35–60.9% have also been reported [40,41,45]. The *hlyA* gene encodes a pore-forming hemolysin which lyses human erythrocytes with subsequent release of iron from heme, a chemical needed for STEC growth and survival in the intestine. Previously, the presence and expression of *hlyA* has been associated with severe STEC disease in humans including HC and HUS [35]. However, STEC that were *hlyA*-negative have also been incriminated in severe disease including bloody diarrhea, HC and HUS, thereby suggesting that the pathogenic role of *hlyA* in STEC remains uncertain [23]. 

Most of the goat STEC were *eaeA-*negative except for the top seven STECs (22.1%) including O157:H7, O26:H2, O111:H8 and O103:H8 and a few (0.7%) non-O157/non-top seven isolates: O71:H14, O108:H25 and O163:H8, in agreement with previous studies which have shown that *eaeA* is not common among goat STEC [40,42,45]. The presence of *eaeA* in goat top seven STEC isolates is of clinical significance as *eaeA* is considered an important STEC adhesin and marker of high virulence and potential to cause severe disease (HC and HUS) in humans [72], especially when accompanied with *stx*2 [23]. However, in some cases, *eaeA*-negative serotypes (O91:H21 and O113:H21) STEC have also been associated with severe disease thereby suggesting that other virulence or unknown host factors may influence disease severity [72,73,74]. The absence of *eaeA* may indicate that goat STEC are less virulent and may also explain why goat STEC are rarely incriminated in human disease worldwide. Of particular interest were *eaeA*-positive goat isolates which belonged to serotypes O103:H8, O71:H14, O108:H25 and O163:H8 but have never been associated with human disease or outbreaks. These isolates will be worth monitoring closely as possession of *eaeA* may be indicative of higher virulence potential and likelihood to cause severe disease in humans. 

## 4. Conclusions

Historically, studies on the presence of STEC in goats are very few compared to cattle which are considered the main STEC reservoir. This study is the first report on the presence of STEC in goats in South Africa. The findings of this study show that goats carry a diverse range of STEC serotypes, some of which have been previously incriminated in mild to severe enteric disease in humans. Collectively, these findings suggest that goats grazing on communal rangeland in South Africa are a reservoir and potential source of STEC for humans in South Africa. Further molecular characterization of goat STEC isolates will be needed in the future to fully assess the virulence potential of goat STEC and capacity to cause disease in humans. In addition, studies that compare STEC isolates from goats and humans will be necessary to fully understand the role played by goats as a source of STEC human disease in South Africa. Data from this study will be useful for understanding the epidemiology of STEC in animals and formulating policies aimed at preventing and controlling zoonotic or foodborne diseases along the food chain. 

## 5. Materials and Methods

### 5.1. Study Population and Sample Collection

**Figure 4:**
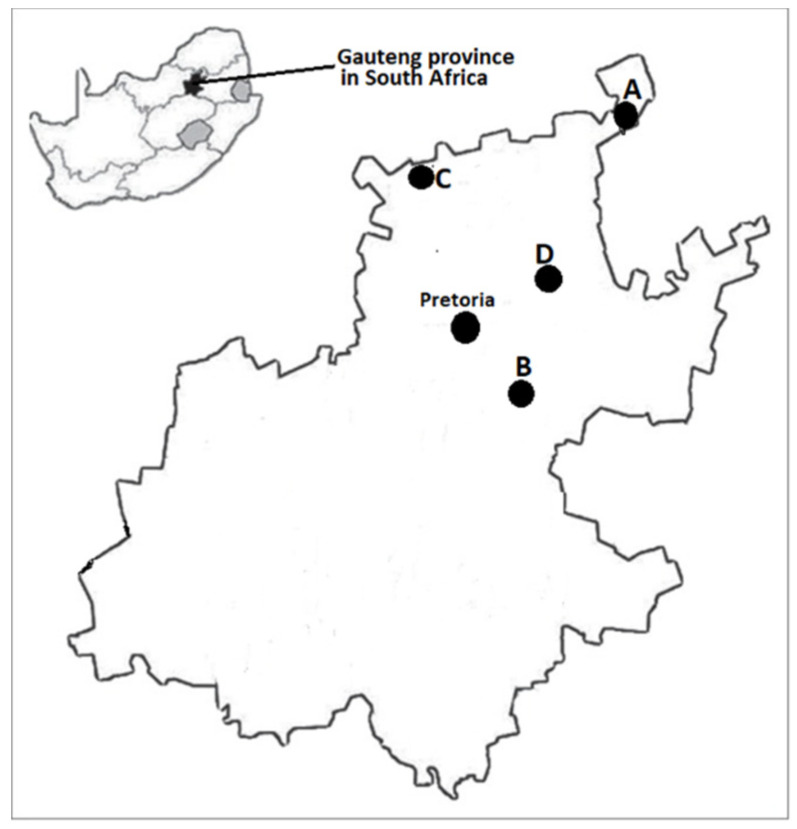
A map of the Gauteng province, South Africa showing the locations of the different herds (A, B, C and D) from which goat fecal samples were obtained.

Goat fecal samples (N = 289) were obtained from four goat herds. The goat herds were located on different communal rangelands in Gauteng province, South Africa. The herds were designated using alphabetical letters: herd A (*n* = 154), herd B (*n* = 43), herd C (*n* = 52) and herd D (*n* = 40). Each herd was visited once. Refer to Figure 4 for a map of the Gauteng province, South Africa showing the locations of the different herds (A, B, C and D) from which goat fecal samples were obtained. Fresh fecal samples were collected by rectal palpation, using a new nitrile examination glove per animal. Samples were placed in sterile specimen containers and transported in a cooler box on ice to the laboratory where they were stored at 4 °C until further processing. Ethical clearance for conducting this research was obtained from the Research Ethics and Animal Ethics Committees of the Faculty of Veterinary Science, University of Pretoria, under approval number REC110-21. 

### 5.2. STEC Culture

Each fecal sample (5 g) was enriched at a 1:10 ratio in EC broth (CM0990, Oxoid, Basingstoke, UK) supplemented with Novobiocin (N1628, Sigma-Aldrich, St. Louis, MO, USA) at 37 °C for 18–24 h. A 100 µL aliquot of the overnight enrichment was spread on Drigalski Lactose agar (CM0531, Oxoid, Basingstoke, UK) and CHROMagar STEC base ST162(B) containing supplement ST162(S) (CHROMagar, Paris, France, http://www.chromagar.com, accessed on 6 April 2022). 

### 5.3. DNA Extraction and STEC Screening

All Drigalski Lactose and CHROMagar STEC agar Petri dishes showing bacterial growth were screened for STEC by PCR [75]. Briefly, a loopful of bacterial colony sweep was collected from each Drigalski Lactose agar and CHROMagar STEC plate showing growth and suspended in 1 mL of FA Buffer (223143, Becton Dickinson and Company, Sparks, MD, USA) [38]. The suspension was homogenised and washed by vortexing, then centrifuged for 5 min. After centrifugation, the supernatant was discarded, and the pellet was re-suspended in FA buffer. After the second wash and centrifugation rounds, the pellet was re-suspended in 500 µL of sterile water, mixed and boiled at 100 °C for 25 min. The boiled preparation was thawed on ice and stored at −20 °C for further processing [38]. A multiplex PCR (mPCR) protocol was used to screen the DNA template for *stx*1, *stx*2, *eaeA* and *hlyA* using previously described cycling parameters and primers [75]. Briefly, each 25 µL PCR reaction mixture contained 2.5 μL of 10X Thermopol reaction buffer, 2.0 μL of 2.5 mM dNTPs (deoxynucleotide triphosphates), 0.25 μL of 100 mM MgCl_2_, 0.6 μL of each primer (10 µM final concentration), 1 U of Taq DNA Polymerase and 5 μL of DNA template. The DNA from *Escherichia coli* O157:H7 strain EDL933 (ATCC 43895) and sterile water were used as positive and negative PCR controls, respectively. All PCR reagents were purchased from New England BioLabs (NEB, Ipswich, MA, USA) except for the primers which were supplied by Inqaba Biotec (Pretoria, South Africa).

### 5.4. STEC Isolation and Identification

For STEC isolation and identification, colony sweeps were collected from Drigalski Lactose agar and CHROMagar plates which were positive for *stx*1 and/or *stx*2 on PCR and streaked onto Drigalski Lactose agar and CHROMagar STEC to obtain single colonies. Five single colonies were purified from each plate and multiplied individually on Luria Bertani agar (REF244520, Becton and Dickinson & Company, Sparks, MD, USA). Once again, DNA was extracted from purified colonies by the boiling method [38]. DNA from each purified colony was screened for *stx*1, *stx*2, *eaeA* and *hlyA* by PCR [75] to verify and confirm the STEC status of each pure colony. Colonies which were positive for *stx*1 and/or *stx*2 were preserved at −80 °C in a bacterial freezing mixture [38] for further O:H serotyping.

### 5.5. STEC Serotyping

All confirmed STEC pure single colonies were serotyped (O:H) by PCR using previously described primers and cycling conditions [57,58,59]. STEC strains which were previously serotyped by traditional serotyping at the National Microbiology Laboratory, Public Health Agency of Canada, Guelph, Ontario, Canada, and the Laboratorio de Referencia de *Escherichia coli* (LREC), Facultad de Veterinaria, Universidad de Santiago de Compostela, Lugo, Spain and a number of *E. coli* O:H types in our collection (unpublished) were also used as positive controls in PCR serotyping assays. Furthermore, the following STEC isolates which were provided by the European Union Reference Laboratory for *Escherichia coli,* Istituto Superiore di Sanità, Rome Italy, were used as positive controls for serotyping the major seven STEC serogroups: STEC-C210-03 (O157), STEC-ED476 (STEC O111), STEC-C1178-04 (STEC O145), STEC-C125-06 (STEC O103) and STEC-ED745 (O26).

## Figures and Tables

**Table 1:** Occurrence of STEC serotypes in the four goat herds (A, B, C and D).

Herds	Herd Occurrence	O Serogroups (N = 34)	Serotypes (N = 63)	Isolates (N = 628)	Number of Goats (N = 289)
A	(75.3%) 116/154	O3 (58)	O3:H2	1	1
			O3:H11	1	1
			O3:H19	1	1
			O3:H21	55	14
		O8 (29)	O8:H14	1	1
			O8:H19	8	2
			O8:H21	5	1
			O8:H49	15	3
		O22 (3)	O22:H8	3	1
		O26 (1)	O26:H2	1	1
		O43 (50)	O43:H2	49	12
			O43:H8	1	1
		O49 (1)	O49:H11	1	1
		O54 (2)	O54:H16	1	1
			O54:H19	1	1
		O64 (1)	O64:H18	1	1
		O71 (3)	O71:H1/12	2	1
			O71:H14	1	1
		O76 (39)	O76:H2	1	1
			O76:H19	38	9
		O103 (99)	O103:H8	98	22
			O103:H56	1	1
		O108 (13)	O108:H19	1	1
			O108:H25	12	3
		O111 (2)	O111:H8	2	1
		O113 (7)	O113:H8	7	3
		O146 (7)	O146:H21	7	2
		O157 (30)	O157:H7	29	7
			O157:H8	1	1
		O163 (1)	O163:H2	1	1
		O175 (2)	O175:H7	1	1
			O175:H19	1	1
		O185 (4)	O185:H8	4	3
		OgN8 (4)	OgN8:H7	4	1
		OgN13 (11)	OgN13:H19	9	1
			OgN13:H10	1	1
			OgN13:H-	1	1
		OgSB9 (7)	OgSB9:H2	1	1
			OgSB9:H19	6	2
		OgX18 (2)	OgX18:H2	2	1
		OgX25 (15)	OgX25:H8	15	3
		ONT (6)	ONT:H18	1	1
			ONT:H19	1	1
			ONT:H26	1	1
			ONT:HNT	3	2
B	(90.6%) 39/43	O5 (3)	O5:H19	3	1
		O6 (14)	O6:H8	1	1
			O6:H21	1	1
			O6:H49	12	6
		O7 (1)	O7:H7	1	1
		O8 (3)	O8:H7	1	1
			O8:H8	2	2
			O8:H14	2	2
		O75 (37)	O75:H8	37	9
		O76 (8)	O76:H19	7	4
			O76:49	1	1
		O79 (1)	O79:H8	1	1
		O125 (1)	O125:H19	1	1
		O132 (1)	O132:H8	1	1
		O159 (1)	O159:H49	1	1
		O163 (1)	O163:H8	1	1
		O176 (5)	O176:H4	5	1
		OgSB9 (6)	OgSB9:H19	5	2
			OgSB9:H21	1	1
		OgX18 (1)	OgX18:H21	1	1
		OgX25 (2)	OgX25:H8	2	1
C	(78.8%) 41/52	O43 (25)	O43:H2	24	7
			O43:H8	1	1
		O76 (4)	O76:H19	4	3
		O113 (1)	O113:H8	1	1
		O146 (11)	O146:H21	11	3
		O157 (2)	O157:H7	1	1
			O157:H29	1	1
		O174 (12)	O174:H8	12	4
		O175 (1)	O175:H21	1	1
		OgX18 (9)	OgX18:H2	9	2
D	(90%) 36/40	O3 (39)	O3:H21	39	9
		O8 (6)	O8:H2	1	1
			O8:H19	5	1
		O76 (1)	O76:H19	1	1
		O146 (13)	O146:H21	13	2
		O159 (1)	O159:H2	1	1
		O174 (4)	O174:H8	4	1
		OgX18 (15)	OgX18:H2	15	4

## Data Availability

Not applicable.

## Supplementary Materials

The following supporting information can be downloaded at: https://www.mdpi.com/article/10.3390/toxins14050353/s1, Table S1: Association between O group and H-type(s) among goat STEC Isolates; Table S2: Goat STEC major virulence factors and gene combinations.
