# Supplementary material for: Occurrence, Serotypes and Virulence Characteristics of Shiga-Toxin-Producing Escherichia coli Isolates from Goats on Communal Rangeland in South Africa
Source: Toxins (Basel). 2022 May 18;14(5):353. doi: 10.3390/toxins14050353 (PMC9147823; doi:10.3390/toxins14050353)
Supplement: Supplementary file 1 [file toxins-14-00353-s001.zip › toxins-1695506-supplementary.pdf]

Supplementary Materials: Occurrence, Serotypes and Virulence Characteristics of Shiga-Toxin-Producing *Escherichia coli* Isolates from Goats on Communal Rangeland in South Africa

Mogaugedi N. Malahlela, Beniamino T. Cenci-Goga, Chris M. Marufu, Thierry Y. Fonkui, Luca Grispoldi, Eric Etter, Alan Kalake and Musafiri Karama

Table S1. Association between O group and H-type(s) among goat STEC Isolates.

O- Group	Associated H- Type
O3	**H2** (1), H11 (1), H19 (1), **H21** (94)
O5	**H19** (3)
O6	H8 (1), H21 (1), **H49** (12)
O7	H7 (1)
O8	**H2** (1), **H7** (1), **H8**(2), **H14** (3), **H19**[d] (13), **H21**[d] (5), **H49** (15)
O22	**H8** (3)
O26	**H2** (1)
O43	**H2** (73), H8 (2)
O49	H11 (1)
O54	H16 (1), H19 (1)
O64	H18 (1)
O71	**H**1/**12** (2), H14 (1)
O75	**H8** (37)
O76	H2 (1), **H19** (50), H49 (1)
O79	H8 (1)
O103	H8 (98), H56 (1)
O108	H19 (1), H25 (12)
O111	**H8** (2)
O113	H8 (8)
O125	**H19** (1)
O132	H8 (1)
O146	**H21** (31)
O157	**H7** (30), **H8** (1), H29 (1)
O159	H2 (1), H49 (1)
O163	H2 (1), H8 (1)
O174	**H8** (16)
O175	H7 (1), H19 (1), **H21** (1)
O176	H4 (5)
O185	H8 (4)
ON8	H7 (4)
ON13	H10 (1), H19 (9), HNT (1)
OSB9	H2 (1), H19 (11), H21(1)
OX18	H2 (26), H21 (1)
OX25	H8 (17)

The numbers in parentheses represent the number of goat isolates.

Serotypes in **bold** have been associated with human disease (Diarrhea, Blood diarrhea, hemorrhagic colitis, Hemolytic Uremic Syndrome) previously (reviewed by Bettelheim and Goldwater. 2019).

Table S2. Goat STEC major virulence factors and gene combinations.

SEROTYPE	No of isolates	*stx1*	*stx2*	*eaeA*	*hlyA*	Gene Combination
O3:H2	1	+	-	-	+	*stx1, hlyA*
O3:H11	1	+	+	-	-	*stx1, stx2*
O3:H19	1	+	+	-	+	*stx1, stx2, hlyA*
O3:H21	38	+	-	-	+	*stx1, hlyA*
O3:H21	39	+	+	-	+	*stx1, stx2, hlyA*
O3:H21	8	+	-	-	-	*stx1*
O3:H21	8	+	+	-	-	*stx1, stx2*
O3:H21	1	-	+	-	-	*stx2*
O5:H19	3	+	+	-	+	*stx1, stx2, hlyA*
O6:H8	1	+	+	-	+	*stx1, stx2, hlyA*
O6:H21	1	-	+	-	-	*stx2, hlyA*
O6:H49	12	-	+	-	-	*stx2*
O7:H7	1	+	+	-	+	*stx1, stx2, hlyA*
O8:H2	1	+	+	-	+	*stx1, stx2, hlyA*
O8:H7	1	-	+	-	-	*stx2*
O8:H8	2	+	+	-	+	*stx1, stx2, hlyA*
O8:H14	1	+	+	-	+	*stx1, stx2, hlyA*
O8:H14	2	-	+	-	-	*stx2*
O8:H19	5	+	+	-	-	*stx1, stx2*
O8:H19	3	+	+	-	+	*stx1, stx2, hlyA*
O8:H19	5	-	+	-	-	*stx2*
O8:H21	5	-	+	-	-	*stx2*
O8:H49	10	-	+	-	+	*stx2, hlyA*
O8:H49	5	-	+	-	-	*stx2*
O22:H8	3	-	+	-	-	*stx2*
O26:H2	1	+	-	+	+	*stx1, eaeA, hlyA*
O43:H2	15	+	+	-	-	*stx1, stx2*
O43:H2	8	+	+	-	+	*stx1, stx2, hlyA*
O43:H2	18	-	+	-	-	*stx2*
O43:H2	29	+	-	-	+	*stx1, hlyA*
O43:H2	3	-	+	-	+	*stx2, hlyA*
O43:H8	1	+	+	-	+	*stx1, stx2, hlyA*
O43:H8	1	+	-	-	+	*stx1, hlyA*
O49:H11	1	+	-	-	+	*stx1, hlyA*
O54:H16	1	+	+	-	-	*stx1, stx2*
O54:H19	1	+	+	-	-	*stx1, stx2*
O64:H18	1	+	-	-	+	*stx1, hlyA*
O71:H1/12	2	-	+	-	+	*stx2, hlyA*
O71:H14	1	-	+	+	+	*stx2, eaeA, hlyA*
O75:H8	33	+	+	-	+	*stx1, stx2, hlyA*
O75:H8	1	+	-	-	-	*stx1*
O75:H8	3	+	+	-	-	*stx1, stx2*
O76:H2	1	+	-	-	+	*stx1, hlyA*
O76:H19	36	+	-	-	+	*stx1, hlyA*

O76:H19	8	+	-	-	-	*stx1*
O76:H19	3	-	+	-	-	*stx2*
O76:H19	1	-	+	-	+	*stx2, hlyA*
O76:H19	2	+	+	-	+	*stx1, stx2, hlyA*
O76:H49	1	+	+	-	+	*stx1, stx2, hlyA*
O79:H8	1	+	+	-	+	*stx1, stx2, hlyA*
O103:H8	96	-	+	+	+	*stx2, eaeA, hlyA*
O103:H8	1	+	-	+	+	*stx1, eaeA, hlyA*
O103:H8	1	+	-	-	+	*stx1, hlyA*
O103:H56	1	+	-	-	+	*stx1, hlyA*
O108:H19	1	+	-	-	-	*stx1*
O108:H25	4	+	-	-	-	*stx1*
O108:H25	4	+	-	+	+	*stx1, eaeA, hlyA*
O108:H25	2	+	-	+	-	*stx1, eaeA,*
O108:H25	2	+	-	-	+	*stx1, hlyA*
O111:H8	2	+	-	+	+	*stx1, eaeA, hlyA*
O113:H8	7	-	+	-	+	*stx2, hlyA*
O113:H8	1	+	+	-	+	*stx1, stx2, hlyA*
O125:H19	1	+	+	-	-	*stx1, stx2*
O132:H8	1	+	-	-	-	*stx1*
O146:H21	16	-	+	-	+	*stx2, hlyA*
O146:H21	10	+	+	-	+	*stx1, stx2, hlyA*
O146:H21	5	+	-	-	-	*stx1*
O157:H7	30	-	+	+	+	*stx2, eaeA,hlyA*
O157:H8	1	-	+	+	+	*stx2, eaeA,hlyA*
O157:H29	1	-	+	-	-	*stx2*
O159:H2	1	+	+	-	+	*stx1, stx2,hlyA*
O159:H49	1	+	+	-	+	*stx1, stx2,hlyA*
O163:H2	1	+	-	-	-	*stx1*
O163:H8	1	+	-	+	+	*stx1, eaeA,hlyA*
O174:H8	13	+	+	-	+	*stx1, stx2, hlyA*
O174:H8	3	+	-	-	+	*stx1, hlyA*
O175:H7	1	+	+	-	+	*stx1, stx2, hlyA*
O175:H19	1	+	-	-	+	*stx1, hlyA*
O175:H21	1	+	+	-	+	*stx1, stx2, hlyA*
O176:H4	5	+	+	-	+	*stx1, stx2, hlyA*
O185:H8	2	+	+	-	-	*stx1, stx2*
O185:H8	2	+	+	-	+	*stx1, stx2, hlyA*
OgN8:H7	4	-	+	-	-	*stx2*
OgN13:H10	1	+	-	-	-	*stx1*
OgN13:H19	8	-	+	-	+	*stx2, hlyA*
OgN13:H19	1	-	+	-	-	*stx2*
OgN13:H-	1	+	-	-	-	*stx1*
OgSB9:H2	1	+	+	-	+	*stx1, stx2, hlyA*
OgSB9:H19	10	+	+	-	+	*stx1, stx2, hlyA*
OgSB9:H19	1	+	-	-	-	*stx1*

OgSB9:H21	1	+	+	-	+	*stx1, stx2, hlyA*
OgX18:H2	2	+	+	-	-	*stx1, stx2*
OgX18:H2	24	+	+	-	+	*stx1, stx2, hlyA*
OgX18:H21	1	+	-	-	-	*stx1*
OgX25:H8	10	-	+	-	+	*stx2, hlyA*
OgX25:H8	7	+	-	-	+	*stx1, hlyA*
ONT:H18	1	+	-	-	+	*stx1, hlyA*
ONT:H19	1	+	-	-	+	*stx1, hlyA*
ONT:H26	1	+	+	-	-	*stx1, stx2,*
ONT:H-	2	+	-	-	+	*stx1, hlyA*
ONT:H-	1	+	+	-	-	*stx1, stx2*
TOTAL	628	381	457	139	490	
% Positive		60.6	72.7	22.1	78.0	

Filename: toxins-1695506-supplementary.docx
Directory: E:\5.17\computation-1719131
Template: C:\Users\MDPI\Desktop\template_supfile.dotx
Title: Title
Subject: A single paragraph of about 200 words maximum. For research articles, abstracts should give a pertinent overview of the work. We strongly encourage authors to use the following style of structured abstracts, but without headings: 1) Background: Place the
Author: MDPI
Keywords: keyword 1; keyword 2; keyword 3. List three to ten pertinent keywords specific to the article, yet reasonably common within the subject discipline.
Comments:
Creation Date: 6/27/2016 8:05:00 PM
Change Number: 24
Last Saved On: 5/17/2022 11:19:00 AM
Last Saved By: MDPI
Total Editing Time: 27 Minutes
Last Printed On: 5/18/2022 10:02:00 AM
As of Last Complete Printing
Number of Pages: 4
Number of Words: 804 (approx.)
Number of Characters: 3,895 (approx.)
